# Mismatch Negativity Predicts Remission and Neurocognitive Function in Individuals at Ultra-High Risk for Psychosis

**DOI:** 10.3389/fpsyt.2020.00770

**Published:** 2020-08-03

**Authors:** Mao Fujioka, Kenji Kirihara, Daisuke Koshiyama, Mariko Tada, Tatsuya Nagai, Kaori Usui, Susumu Morita, Shintaro Kawakami, Kentaro Morita, Yoshihiro Satomura, Shinsuke Koike, Motomu Suga, Tsuyoshi Araki, Kiyoto Kasai

**Affiliations:** ^1^ Department of Neuropsychiatry, Graduate School of Medicine, The University of Tokyo, Tokyo, Japan; ^2^ Department of Psychiatry, University of California San Diego, La Jolla, CA, United States; ^3^ The International Research Center for Neurointelligence (WPI-IRCN), University of Tokyo Institutes for Advanced Study (UTIAS), The University of Tokyo, Tokyo, Japan; ^4^ Department of Psychiatry, Kawamuro Memorial Hospital, Joetsu, Japan; ^5^ Department of Rehabilitation, Graduate School of Medicine, The University of Tokyo, Tokyo, Japan; ^6^ University of Tokyo Institute for Diversity and Adaptation of Human Mind (UTIDAHM), The University of Tokyo, Tokyo, Japan; ^7^ Center for Evolutionary Cognitive Sciences, Graduate School of Art and Sciences, The University of Tokyo, Tokyo, Japan; ^8^ UTokyo Center for Integrative Science of Human Behaviour (CiSHuB), The University of Tokyo, Tokyo, Japan; ^9^ Graduate School of Clinical Psychology, Teikyo Heisei University, Tokyo, Japan

**Keywords:** mismatch negativity, ultra-high risk for psychosis, longitudinal study, remission, neurocognitive function

## Abstract

**Background:**

In the early intervention in psychosis, ultra-high risk (UHR) criteria have been used to identify individuals who are prone to develop psychosis. Although the transition rate to psychosis in individuals at UHR is 10% to 30% within several years, some individuals at UHR present with poor prognoses even without transition occurring. Therefore, it is important to identify biomarkers for predicting the prognosis of individuals at UHR, regardless of transition. We investigated whether mismatch negativity (MMN) in response to both duration deviant stimuli (dMMN) and frequency deviant stimuli (fMMN) could predict prognosis, including remission and neurocognitive function in individuals at UHR.

**Materials and Methods:**

Individuals at UHR (n = 24) and healthy controls (HC; n = 18) participated in this study. In an auditory oddball paradigm, both dMMN and fMMN were measured at baseline. Remission and neurocognitive function after > 180 days were examined in the UHR group. Remission from UHR was defined as functional and symptomatic improvement using the Global Assessment of Functioning (GAF) score and Scale of Prodromal Symptoms (SOPS) positive subscales. Neurocognitive function was measured using the Brief Assessment of Cognition in Schizophrenia (BACS). We examined differences in MMN amplitude at baseline between those who achieved remission (remitters) and those who did not (non-remitters). Multiple regression analyses were performed to identify predictors for functioning, positive symptoms, and neurocognitive function.

**Results:**

Compared with the HC group, the UHR group had a significantly attenuated dMMN amplitude (*p* = 0.003). In the UHR group, GAF scores significantly improved during the follow-up period (mean value 47.1 to 55.5, *p* = 0.004). The dMMN amplitude at baseline was significantly larger in the remitter (n = 6) than in the non-remitter group (n = 18) (*p* = 0.039). The total SOPS positive subscale scores and fMMN amplitude at baseline could predict BACS attention subscore at the follow-up point (SOPS positive subscales, *p* = 0.030; fMMN, *p* = 0.041).

**Conclusion:**

Our findings indicate that dMMN and fMMN predicted remission and neurocognitive function, respectively, in individuals at UHR, which suggests that there are both promising biomarker candidates for predicting prognosis in individuals at UHR.

## Introduction

Early intervention for individuals with schizophrenia is important, as the duration of untreated psychosis is known to predict the outcomes of schizophrenia (1). Furthermore, detection of schizophrenia before the onset of psychosis may lead to its prevention. However, identifying individuals who will develop psychosis at a later time is difficult at the prodromal stage because the symptoms are not disease-specific. Consequently, the ultra-high risk for psychosis (UHR) criteria, which include the brief intermittent psychotic syndrome (BIPS), attenuated positive symptom syndrome (APS), and genetic risk and deterioration syndrome (GRDS) assessments were developed (2, 3) and have been used (4) to identify individuals at high risk for psychosis.

Previous studies that have investigated the prognosis of individuals at UHR have mostly focused on the transition to psychosis. When the concept of UHR criteria was introduced, the transition rate in the first year was 40% to 50% (2, 3). However, a recent meta-analysis showed a decreasing trend of 22% and 36% at the one-year and three-year follow-up time-points, respectively (4). Although many individuals at UHR do not transition to psychosis (non-converters), their prognosis is not necessarily good. A meta-analysis by Simon et al. reported that 73% of individuals at UHR did not transition to psychosis within 2 years of follow-up, and the percentage of non-converters who achieved remission from UHR status was 46% (5). In a six-year follow-up study of individuals who met the UHR or basic symptoms (BS) criteria, which is another tool for risk assessment for psychosis, approximately 40% of the participants achieved full remission from UHR symptoms (6). Further, the functional prognosis is also poor. Another six-year, longitudinal, structural magnetic resonance imaging study of UHR or BS individuals reported that more than half of the participants had poor functional outcomes with a modified Global Assessment of Functioning scale score of < 65 (7). A longitudinal study examining the natural history of 111 non-converted medication-naive individuals at UHR reported an improvement of baseline social and role functions over the course of 2 years. However, these functions were still significantly lower compared to those of nonpsychiatric participants (8). In summary, the prognosis of individuals at UHR is symptomatically and functionally poor, even if they do not transition to psychosis. Therefore, it is important to investigate and identify biomarkers for predicting prognoses and allowing for early intervention for individuals at UHR, regardless of whether they transition to psychosis.

To date, various indices have been studied to predict the transition to psychosis in individuals at UHR. Mismatch negativity (MMN), a negative component of event-related potentials (ERP) elicited by infrequent deviant stimuli occurring within a series of frequent standard stimuli, is considered a promising biomarker among other ERP components (9). In schizophrenia, the MMN amplitude decreases with a large effect size (10, 11). Moreover, MMN deficiency, which reflects functional impairment of N-methyl-D-aspartate receptors (12, 13), is associated with neurocognitive (14, 15) and functional decline (16, 17).

Although MMN is a promising biomarker for predicting the transition to psychosis (18, 19), it remains unclear whether MMN predicts the prognosis of individuals at UHR, regardless of transition to psychosis. Kim et al. showed that the amplitude of MMN in response to duration deviant stimuli (dMMN) predicted later remission, improvement in attenuated positive symptoms, and functional recovery (20). However, longitudinal studies using other deviant stimuli, such as intensity and frequency, have not been reported. Because the association between MMN to frequency deviant stimuli (fMMN) and global functioning differs from that between dMMN and global functioning (21), both fMMN and dMMN should be investigated. Further, neurocognitive function is also important since it mediates the association between MMN and functional outcomes in chronic schizophrenia (22). Therefore, the aim of this study was to investigate whether both dMMN and fMMN can predict future remission and neurocognitive function in individuals at UHR for psychosis.

## Materials and Methods

### Participants

This study was performed as part of our multimodal research project to investigate biomarkers for psychosis [IN-STEP: the Integrative Neuroimaging Studies in Schizophrenia Targeting for Early Intervention and Prevention (23)]. At study enrollment, 39 out of 53 participants recruited to IN-STEP as individuals at UHR underwent EEG recording; among them, 24 underwent follow up. The current study enrolled 24 individuals at UHR and 18 healthy controls (HC). Among these 42 participants, 41 had participated in our previous ERP studies (21, 24–26).

Individuals at UHR were recruited from the outpatient and inpatient units at the University of Tokyo Hospital while HC participants were recruited through advertisements at several universities in Tokyo. The inclusion criteria of individuals at UHR were: aged 12–30 years; a history of antipsychotic ≤ 16 cumulative weeks at the enrollment time in the IN-STEP project; and confirmed as being at UHR using the Structured Interview for Prodromal Symptoms (SIPS) (27, 28). The inclusion criteria of HCs were: aged 12–40 years; no history of psychiatric disease [confirmed using the Japanese version of the Mini International Neuropsychiatric Interview (29)]; and no family history of first-degree relatives diagnosed with an axis I disorder based on the criteria of the Diagnostic and Statistical Manual of Mental Disorders, Fourth Edition (DSM-IV) (30). The exclusion criteria for all participants were any neurological illness, traumatic brain injury with cognitive consequences or loss of consciousness for > 5 minutes, a history of electroconvulsive therapy, a low premorbid intelligence quotient [IQ; < 70 as estimated using the Japanese version of the National Adult Reading Test (31, 32)], previous alcohol or substance abuse or addiction, and hearing impairment revealed by audiometer testing in both ears at a 30 dB sound pressure level and a tone frequency of 1,000 Hz and 40 dB at 4,000 Hz. Inclusion and exclusion criteria have been described in greater detail by Koike et al. (23). Written informed consent in accordance with the Declaration of Helsinki was obtained from each participant before enrolment in the study. For participants aged < 20 years, written informed assent and consent were obtained from the participant and his/her parents, respectively. This study was approved by the Research Ethics Committee of the Faculty of Medicine of the University of Tokyo (approval no. 629 and 2226).

At baseline, all of the participants underwent electroencephalogram (EEG) recording, and their global and neurocognitive functions were assessed by the Global Assessment of Functioning (GAF) (30) and the Brief Assessment of Cognition in Schizophrenia (BACS) (33, 34) scales, respectively. For individuals at UHR, psychotic symptoms were also measured by the Scale of Prodromal Symptoms (SOPS) positive subscale included in the SIPS assessment at baseline (Time 1). Further, individuals at UHR underwent assessment at > 180 days after baseline (Time 2) to determine prognosis, including the transition to psychosis, using the GAF and BACS scales, as well as the SIPS/SOPS (individuals who had transitioned to psychosis were not assessed by SOPS at Time 2). Remission from UHR status was defined by a score of ≥ 61 on the GAF and ≤ 2 on all SOPS positive subscales at the last follow-up time-point without transitioning to psychosis, according to previous studies (20, 35). For individuals at UHR, the prescribed antipsychotic drug doses were converted to chlorpromazine equivalent doses (36). Moreover, we examined the duration of untreated prodromal psychosis, which is the period between the appearance of the first prodromal symptom and the first hospital visit.

### Stimuli and Procedure

Two auditory oddball paradigms using duration and frequency deviant stimuli were employed. For dMMN, 2,000 stimuli consisting of 90% standard tones (1,000 Hz, 50 ms) and 10% deviant tones (1,000 Hz, 100 ms) were used. For fMMN, 2,000 stimuli consisting of 90% standard tones (1,000 Hz, 50 ms) and 10% deviant tones (1,200 Hz, 50 ms) were used. The order of the two paradigms was counterbalanced across participants. All stimuli were presented binaurally through earphones while participants sat watching a silent cartoon. The auditory parameters were delivered at an 80-dB sound pressure level, 1 ms rise/fall time, and 500 ms stimulus-onset asynchrony.

### EEG Recording and Analyses

EEG data were recorded using a 64-channel Geodesic EEG System (Electrical Geodesics Inc., Eugene, OR). The electrodes were referenced to the vertex with the impedances being maintained < 50 kΩ. The sampling rate was set at 500 Hz with the analog filter bandpass set at 0.1 to 100 Hz.

**Figure 1:**
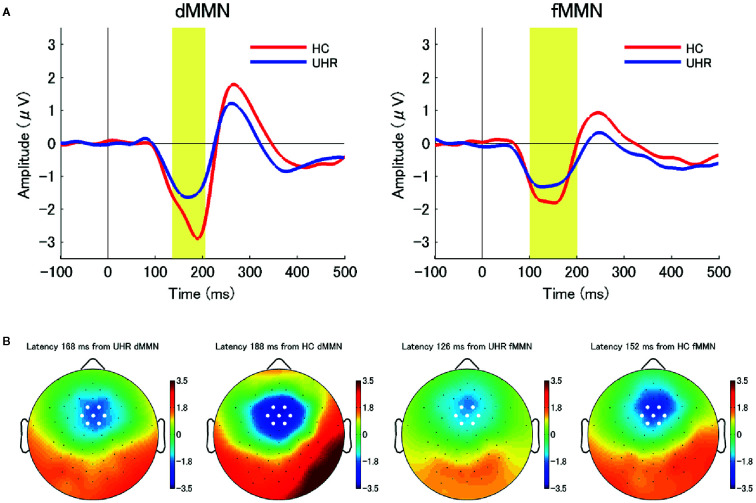
**(A)** The average waveforms for duration mismatch negativity (dMMN; left) and frequency mismatch negativity (fMMN; right) at the seven electrodes around the frontocentral electrode (FCz) in individuals at ultra-high risk for psychosis (UHR; blue line) and healthy control (HC) participants (red line). Between-group differences in the dMMN amplitudes were significant at the.01 level. **(B)** Two-dimensional topographic maps at the latency of peak amplitudes of dMMN (left) and fMMN (right) in both groups. The latency of peak amplitudes were 168 ms (dMMN, UHR), 188 ms (dMMN, HC), 126 ms (fMMN, UHR), and 152 ms (fMMN, HC). White circles represent the seven electrodes around the FCz.

**Figure 2:**
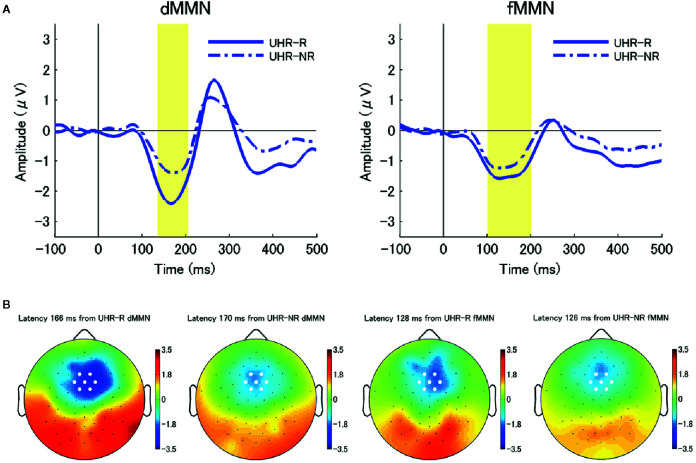
**(A)** The average waveforms for duration mismatch negativity (dMMN; left) and frequency mismatch negativity (fMMN; right) at the seven electrodes around the frontocentral electrode (FCz) at baseline (Time 1) in individuals at ultra-high risk for psychosis (UHR) who achieved remission (remitters) (UHR-R; remitted at Time 2; N = 6; blue solid line) and those who did not (non-remitters) (UHR-NR; not remitted at Time 2; N = 18; blue chain line). There were significant between-subgroup differences in the dMMN amplitudes at the.05 level. **(B)** Two-dimensional topographic maps of the latency of peak amplitudes of dMMN (left) and fMMN (right) in both subgroups. The latency of the peak amplitudes were 166 ms (dMMN, UHR-R), 170 ms (dMMN, UHR-NR), 128 ms (fMMN, UHR-R), and 126 ms (fMMN, UHR-NR), respectively. White circles represent the seven electrodes around the FCz.

The data were analyzed with EEGLAB, which is an open-source toolbox for EEG analysis (37). EEG signals at each electrode were re-referenced using an average reference and digitally filtered at 0.1–20 Hz. Epochs were extracted from −100 to 500 ms relative to the stimulus onset; further, the baseline was corrected by subtracting the mean amplitude from −100 to 0 ms. Eyeblink artifacts were corrected through independent component analysis. Epochs exceeding ± 100 μV at each electrode were excluded. ERP waveforms for both standard and deviant stimuli were obtained through across-trial averaging. The MMN waveform was obtained as the difference in the average waveforms between the standard and deviant stimuli. We computed the dMMN and fMMN amplitudes as the mean amplitudes from 135 to 205 ms and from 100 to 200 ms post-stimulus, respectively, as we previously reported (21, 24–26).To analyze the MMN amplitude, we selected seven electrodes around the frontocentral electrode FCz (Geodesic Sensor Net (GSN) numbers: 3, 4, 5, 8, 9, 55, and 58) where the largest MMN amplitude was obtained (the selected electrodes were shown as white circles in the topographic maps in **Figures 1** and **2**). The average amplitude of these seven electrodes was used as the MMN amplitude for each participant.

### Statistical Analysis

IBM® Statistical Package for the Social Sciences (SPSS®) Statistics version 25 (IBM Corp., New York, USA) was used for all statistical analyses. Chi-squared tests and t-tests were used for comparing categorical and continuous variables of demographic characteristics between the UHR and HC groups at Time 1. When the variances were not equal, Welch’s t-test was used instead of an independent t-test. For individuals at UHR, clinical characteristics between Time 1 and Time 2 were compared using paired t-tests to assess longitudinal changes. We compared demographic characteristics, clinical characteristics, and MMN amplitude at Time 1 between the 24 and 15 individuals at UHR who were followed and not followed up at Time 2, respectively.

Baseline demographic and clinical characteristics, including dMMN and fMMN amplitudes, were compared between the UHR-R and UHR-NR subgroups using Fisher’s exact tests and Mann-Whitney U-tests for categorical and continuous variables, respectively. To evaluate whether baseline MMN amplitudes could predict functional, symptomatic, and neurocognitive prognosis, we performed a multiple regression analysis using the stepwise selection method. Here, we used the following independent variables: both dMMN and fMMN amplitudes at baseline; demographic characteristics, including sex, age at baseline, and premorbid IQ; GAF score and total SOPS positive subscale score at baseline; follow-up period; and antipsychotics use. We adopted the GAF score; total SOPS positive subscale scores; and each BACS subscore, including the composite score, at Time 2 as the dependent variables. Statistical significance was set at .05.

## Results

### Demographic and Clinical Characteristics

The demographic and clinical characteristics at baseline (Time 1) are shown in **Table 1**. Individuals at UHR were categorized based on whether they met the criteria for BIPS, APS, and GRDS. The number individuals at UHR who met the aforementioned criteria were: 2 (8%) for BIPS only; 16 (67%) for APS only; 1 (4%) for GRDS only; and 5 (21%) for APS + GRDS. None of the individuals at UHR met the remission criteria at baseline. GAF scores were significantly different between the UHR and HC groups (t_28.9_ = −20.46, *p* < 0.001). Moreover, the dMMN amplitude at baseline in the UHR group was significantly attenuated compared to that in the HC group (t_40_ = 3.13, *p* = 0.003) while there was no significant between-group difference in the fMMN amplitudes. **Figure 1** shows the grand-average waveforms and two-dimensional topographic maps of both dMMN and fMMN at baseline in both groups. There was no significant difference in the demographic or clinical characteristics at baseline between individuals with and without follow up at Time 2. The **Supplementary Table** shows the comparison between individuals at UHR with and without follow-up.

**Table 1:** Demographic and clinical characteristics of the participants at baseline (Time 1).

	UHR (N = 24)	HC (N = 18)	Statistics	
Sex (Male/Female)^a^	12/12	10/8	χ^2^ = 0.13, df = 1	p = 0.721
Age (years)^b,c^	20.4 (3.7) 14–28	21.9 (3.6) 16–30	t_40_ = −1.29	p = 0.204
Education (years)^b^	12.7 (2.6)	13.8 (2.5)	t_40_ = −1.35	p = 0.186
Premorbid IQ^b^	105.9 (8.8)	107.1 (9.1)	t_40_ = −0.43	p = 0.667
GAF^d^	47.1 (9.4)	88.8 (3.0)	t_28.9_ = −20.46	p < 0.001^***^
BACS (z score)				
Composite^d,e^	−0.28 (0.91)	0.03 (0.53)	t_37.8_ = −1.41	p = 0.167
Verbal memory^b^	−0.33 (1.20)	−0.10 (0.93)	t_40_ = −0.68	p = 0.502
Working memory^b^	−0.33 (1.20)	0.27 (0.86)	t_40_ = −1.79	p = 0.081
Motor speed^b,e^	−0.77 (1.56)	0.36 (1.23)	t_39_ = −0.91	p = 0.367
Verbal fluency^b^	0.00 (1.48)	0.20 (1.45)	t_40_ = −0.44	p = 0.661
Attention^b^	−0.08 (1.18)	0.35 (0.67)	t_40_ = −1.40	p = 0.169
Executive function^b^	−0.20 (1.26)	0.10 (0.63)	t_40_ = −0.91	p = 0.366
dMMN amplitude (μV)^b^	−1.49 (0.85)	−2.36 (0.94)	t_40_ = 3.13	p = 0.003^**^
fMMN amplitude (μV)^d^	−1.15 (0.57)	−1.43 (0.95)	t_26.0_ = 1.10	p = 0.283

All values except for sex are shown as means (standard deviation). ^**^The mean difference is significant at the .01 level; ^***^The mean difference is significant at the .001 level.

UHR, ultra-high risk for psychosis; HC, healthy control; IQ, intelligence quotient; GAF, Global Assessment of Functioning; BACS, Brief Assessment of Cognition in Schizophrenia; dMMN, duration mismatch negativity; fMMN, frequency mismatch negativity.

^a^Chi-square tests used for statistical comparisons.

^b^Independent t-tests used for statistical comparisons.

^c^The range is described in the lower row.

^d^Welch’s t-test used for statistical comparisons.

^e^One HC participant had a missing BACS subscore related to motor speed.

### Longitudinal Changes in Clinical Characteristics

**Table 2** shows the longitudinal changes in clinical characteristics in individuals at UHR. The average follow-up time was 604 days (standard deviation: 297 days; range: 185–1,133 days). Further, 3 out of the 24 individuals at UHR developed psychosis during the follow-up period, all of whom were diagnosed with schizophrenia based on the DSM-IV criteria. GAF scores improved over time, even for those individuals who transitioned to psychosis (mean value: 47.1 to 55.5, *p* = 0.004). Further, the SOPS positive subscale scores improved among non-converters (mean value of total SOPS positive subscales: 9.4 to 5.2, *p* < 0.001). Six individuals met the remission criteria at Time 2. There was an improvement in the verbal memory (mean value: −0.33 to 0.25, *p* = 0.002); however, there were no improvements in the other BACS subscores, including the composite score. There were no significant differences in the chlorpromazine-equivalent doses of antipsychotic drugs between Time 1 and Time 2.

**Table 2:** Longitudinal changes in clinical characteristics in individuals at UHR.

	Time 1	Time 2	Statistics	
GAF^a^	47.1 (9.4)	55.5 (12.4)	t_23_ = −3.15	p = 0.004^**^
Total SOPS positive subscales^a,b^	9.4 (3.6)	5.2 (2.9)	t_20_ = 4.68	p < 0.001^***^
BACS (z score)				
Composite^a^	−0.28 (0.91)	−0.09 (0.71)	t_23_ = −1.63	p = 0.117
Verbal memory^a^	−0.33 (1.20)	0.25 (0.95)	t_23_ = −3.54	p = 0.002^**^
Working memory^a^	−0.33 (1.20)	−0.24 (1.10)	t_23_ = −0.45	p = 0.655
Motor speed^a^	−0.77 (1.56)	−0.60 (1.18)	t_23_ = −0.62	p = 0.543
Verbal fluency^a^	0.00 (1.48)	−0.25 (1.20)	t_23_ = 1.29	p = 0.211
Attention^a^	−0.08 (1.18)	0.01 (1.23)	t_23_ = −0.43	p = 0.671
Executive function^a^	−0.20 (1.26)	0.29 (0.99)	t_23_ = −1.63	p = 0.116
Antipsychotics (mg/day)^a,c^	113.6 (146.7)	204.6 (359.1)	t_23_ = −1.32	p = 0.199

All values are shown as means (standard deviation). ^**^The mean difference is significant at the .01 level; ^***^The mean difference is significant at the .001 level.

UHR, ultra-high risk for psychosis; GAF, Global Assessment of Functioning; SOPS, Scale of Prodromal Symptoms; BACS, Brief Assessment of Cognition in Schizophrenia; CP, Chlorpromazine.

^a^Paired t-test used for statistical comparison.

^b^Except for three individuals who had progressed to psychosis.

^c^Reported as chlorpromazine equivalent dose.

### Relationship Between Baseline MMN Amplitudes and Prognosis

The dMMN amplitude at baseline were significantly larger in the remitter group (UHR-R; n = 6) than in the non-remitter group (UHR-NR; n = 18) (*p* = 0.039). However, there were no significant differences in the fMMN amplitude between the UHR-R and UHR-NR subgroups (*p* = 0.096). **Figure 2** shows the grand-average waveforms and two-dimensional topographic maps for each group. **Table 3** shows the between-subgroup differences in the baseline demographic and clinical characteristics, as well as MMN amplitudes. There was no between-subgroup difference in the GAF, SOPS positive subscale, or BACS scores, as well as the chlorpromazine-equivalent doses of antipsychotic drugs. The UHR-R group exhibited a longer follow-up period, had a younger mean age, and fewer years of education at baseline compared to the UHR-NR subgroup.

**Table 3:** Baseline demographic and clinical characteristics of the UHR-R and UHR-NR subgroups.

	UHR-R (N = 6)	UHR-NR (N = 18)	Statistics	
Sex (male/female)^a^	3/3	9/9		p = 1.000
Follow-up period (days)^b^	899.5 (179.7)	505.8 (263.1)	U_6,18_ = 12.0	p = 0.005^**^
Age (years)^b^	17.7 (2.9)	21.3 (3.5)	U_6,18_ = 22.0	p = 0.031^*^
Education (years)^b^	10.8 (2.6)	13.3 (2.3)	U_6,18_ = 23.5	p = 0.040^*^
Premorbid IQ^b^	101.3 (9.2)	107.4 (8.4)	U_6,18_ = 30.5	p = 0.114
DUPP (days)^b,c^	312.3 (437.9)	307.4 (500.3)	U_6,16_ = 44.0	p = 0.768
GAF^b^	50.8 (6.3)	45.8 (10.0)	U_6,18_ = 32.5	p = 0.150
Total SOPS positive subscales^b^	8.7 (3.7)	10.6 (4.0)	U_6,18_ = 39.5	p = 0.330
BACS (z score)				
Composite^b^	0.10 (0.83)	−0.41 (0.92)	U_6,18_ = 33.0	p = 0.162
Verbal memory^b^	0.08 (0.87)	−0.46 (1.28)	U_6,18_ = 42.0	p = 0.423
Working memory^b^	−0.51 (1.26)	−0.27 (1.22)	U_6,18_ = 51.5	p = 0.867
Motor speed^b^	−0.21 (1.55)	−0.96 (1.56)	U_6,18_ = 36.5	p = 0.243
Verbal fluency^b^	0.56 (1.86)	−0.19 (1.34)	U_6,18_ = 43.0	p = 0.463
Attention^b^	0.40 (1.04)	−0.24 (1.21)	U_6,18_ = 35.0	p = 0.205
Executive function^b^	0.27 (0.68)	−0.36 (1.38)	U_6,18_ = 36.0	p = 0.228
Antipsychotics (mg/day)^b,d^	66.7 (108.0)	129.3 (157.0)	U_6,18_ = 42.0	p = 0.390
dMMN amplitude (μV)^b^	−2.13 (0.94)	−1.27 (0.73)	U_6,18_ = 23.0	p = 0.039^*^
fMMN amplitude (μV)^b^	−1.43 (0.55)	−1.06 (0.56)	U_6,18_ = 29.0	p = 0.096

All values except for sex are shown as means (standard deviation). ^*^The mean difference is significant at the .05 level; ^**^The mean difference is significant at the .01 level.

UHR, ultra-high risk for psychosis; UHR-R, UHR remitters; UHR-NR, UHR non-remitters; IQ, intelligence quotient; DUPP, duration of untreated prodromal psychosis; GAF, Global Assessment of Functioning; SOPS, Scale of Prodromal Symptoms; BACS, Brief Assessment of Cognition in Schizophrenia; CP, Chlorpromazine; dMMN, duration mismatch negativity; fMMN, frequency mismatch negativity.

^a^Fisher’s exact test used for statistical comparison.

^b^Mann-Whitney U-test used for statistical comparison.

^c^Two participants in the UHR-NR participant have missing DUPP data.

^d^Reported as chlorpromazine equivalent dose.

**Figure 3:**
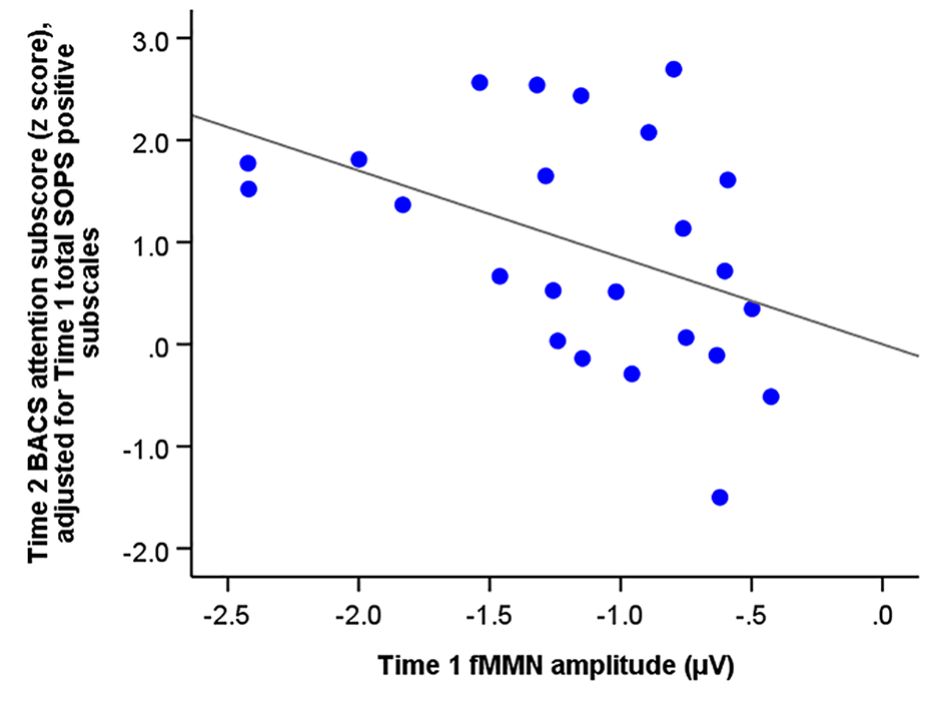
The correlation between the amplitude of frequency mismatch negativity (fMMN) of individuals at ultra-high risk for psychosis at baseline (Time 1) and the Brief Assessment of Cognition (BACS) attention subscore (z score) at Time 2 adjusted for total SOPS positive subscales at baseline (Time 1).

Multiple regression analysis revealed that the total SOPS positive subscales and fMMN amplitude at baseline predicted the BACS attention subscores at follow-up (F_2,21_ = 4.98, R^2^ = 0.32, *p* = 0.017; SOPS positive subscales, beta = 0.13 [95% CI, 0.01 to 0.25], standardized beta = 0.42, t = 2.33, *p* = 0.030; fMMN, beta = −0.85 [95% CI, −1.66 to −0.04], standardized beta = −0.39, t = −2.18, *p* = 0.041). **Figure 3** shows the association between fMMN at baseline and BACS attention subscore at the follow-up point, which was adjusted for total SOPS subscales at baseline.

## Discussion

This study assessed whether baseline dMMN and fMMN amplitudes could predict the prognosis of individuals at UHR, regardless of whether they transitioned to psychosis. There were significantly larger dMMN amplitudes at baseline in individuals at UHR who achieved remission compared with those who did not. Further, multiple regression analysis revealed that fMMN amplitude at baseline predicted the BACS attention score at the follow-up point.

### Functioning and Neurocognitive Function in UHR

Compared with the HC group, the UHR group showed significantly reduced the GAF score; however, there was no between-group difference in the BACS score. There have been numerous reports of impaired functioning in individuals at UHR (8, 38). Moreover, a meta-analysis by Fusar-Poli et al. reported reduced functioning in the UHR group by around three in the effect size compared to HC participants (39). Further, neurocognitive function is reduced in various domains; however, the effect size is small with a maximum of around 0.5 (40). We found that neurocognitive functions were not significantly reduced in UHR. The effect sizes of neurocognitive impairments in UHR range from 0.14 to 0.79 (Cohen’s *d*). Therefore, impaired functioning in individuals at UHR could be attributed to clinical symptoms rather than neurocognitive impairments. Specifically, improvements in positive and negative symptoms contributed to improved social and role functioning in UHR, respectively (41). Further, depression and anxiety, which are often comorbid with UHR (8), may influence functioning. However, it was previously reported that neurocognitive function could predict future functioning in UHR (38, 42, 43). Since usual treatments, including pharmacotherapy and psychotherapy, can improve clinical symptoms but not neurocognitive function, neurocognitive impairments could limit functioning improvement in individuals at UHR.

### Future Remission Prediction by Duration MMN

Compared with the UHR-NR group, the UHR-R group showed a large dMMN, but not fMMN. This indicates that dMMN may predict future remission in UHR, which is consistent with previous reports that dMMN can predict future remission in individuals at UHR (20). In addition, these findings indicate that prediction of future remission in individuals at UHR by MMN is dependent on deviant type.

Previous cross-sectional studies have reported an association of dMMN with functioning in schizophrenia (21, 44) and UHR (21, 45). Several studies (21, 45, 46), including this study, have reported reduced dMMN amplitude compared with HC. Moreover, neural circuits underlying dMMN could be impaired in UHR, which may not only affect current functioning but also future remission. For example, Kim et al. reported reduced dMMN current source density in the right frontal cortex and functional disconnection between the temporal and frontal cortices in UHR (46). Given the previous reports of MMN generators in multiple cortical sources (47) and connectivity among the cortical sources (48), these neural circuits could attribute to reduced dMMN and prediction of future remission in UHR. However, there is a need for further studies to clarify neural circuits underlying reduced dMMN and future remission prediction in UHR.

### Prediction of Future Neurocognitive Function by Frequency MMN

We found that the fMMN amplitude along with the baseline positive symptoms predicted future neurocognitive function of attention. Although individuals at UHR presented intact fMMN as a group, inter-individual differences in fMMN could affect future neurocognitive function. To our knowledge, this is the first study to report that fMMN can predict the UHR prognosis.

In schizophrenia, impaired early auditory information processing, as indicated by reduced MMN, could affect neurocognitive deficits, which results in subsequent poor functional outcomes (22). However, previous cross-sectional studies have reported inconsistent findings regarding the correlations between MMN and neurocognitive function in UHR. Higuchi et al. reported a correlation between dMMN and verbal fluency in UHR (49). Moreover, Carrión et al. reported a correlation between fMMN and processing speed across individuals at UHR and HCs (45). A previous cross-sectional study of our group reported no significant correlation of either dMMN or fMMN with neurocognitive function in UHR (21). In this study, there was no correlation of MMN amplitudes with each BACS subscore at baseline. These current findings indicate that fMMN may be associated with future, but not current, neurocognitive function.

Contrastingly, the attention neurocognition domain, which we found to be associated with fMMN, has been found to predict functioning. Sawada et al. reported that BACS attention subscores could predict future modified GAF scores in individuals at UHR who do not develop psychosis (43). The attention subdomain of the BACS is measured using the symbol coding task. Further, the processing speed, which is indicated by similar digit symbol-coding subtests and Trail Making Test, could predict social functioning (38, 42). Therefore, fMMN may affect functional prognosis through neurocognitive function. However, we could not perform, for example, structural equation modeling to investigate the fMMN effect on the future functional outcome through neurocognitive function given the small sample size. There is a need for further studies to clarify this.

### Limitations

This study had several limitations. First, we adopted a naturalistic design and did not control for the duration of the follow-up period or any medications used by the participants. The inter-individual difference in the follow-up period and medication use could have biased and affected the results. The impact of various psychotropic drugs on MMN remains unclear (50); however, antipsychotics and benzodiazepines have been shown not to affect MMN (51, 52). Second, some of the individuals at UHR had relatively short follow-up periods. Although the mean follow-up period was around 2 years, the follow-up period was < 1 year in 6 out of the 24 individuals at UHR. Following up on these 6 individuals at UHR for longer periods could have changed their prognosis. Finally, there were unavailable MMN data upon follow-up. Only 10 out of the 24 individuals at UHR had MMN data at the follow-up point; moreover, the sample size was insufficient for analyses. The availability of MMN data at both baseline and follow-up points could contribute to a more comprehensive understanding of the longitudinal association between MMN and prognosis.

## Conclusion

In conclusion, we observed an association between future remission and dMMN in individuals at UHR. Moreover, we found that fMMN predicted neurocognitive function in UHR. These findings suggest that both dMMN and fMMN could be candidate biomarkers for predicting the prognosis of individuals at UHR.

## Data Availability Statement

The raw data supporting the conclusions of this article may be available upon request only if the ethical committee of Faculty of Medicine, the University of Tokyo approves it.

## Ethics Statement

The studies involving human participants were reviewed and approved by the Research Ethics Committee of the Faculty of Medicine, The University of Tokyo. Written informed consent to participate in this study was provided by the participants or their parents.

## Author Contributions

KKi, DK, MT, and TN collected the data. MF, KKi, DK, MT, TN, and KU analyzed the data. MF, KKi, DK, MT, and KU interpreted the results. SKo, MS, TA, and KKa designed the study. KKa supervised all aspects of data collection, analysis, and interpretation. MF, KKi, and KKa wrote the first draft of the manuscript. All the authors commented on the manuscript. All authors contributed to the article and approved the submitted version.

## Funding

This study was supported in part by the Japan Agency for Medical Research and development, AMED (grant JP20dm0207069), Japan Society for the Promotion of Science, JSPS KAKENHI (grants JP16H06395, JP16H06399, and JP16K21720), Takeda Science Foundation, UTokyo Center for Integrative Science of Human Behavior (CiSHuB), and the International Research Center for Neurointelligence (WPIIRCN) at The University of Tokyo Institutes for Advanced Study (UTIAS).

## Conflict of Interest

The authors declare that the research was conducted in the absence of any commercial or financial relationships that could be construed as a potential conflict of interest.
