# Supplementary material for: Mismatch Negativity Predicts Remission and Neurocognitive Function in Individuals at Ultra-High Risk for Psychosis
Source: Front Psychiatry. 2020 Aug 3;11:770. doi: 10.3389/fpsyt.2020.00770 (PMC7416637; doi:10.3389/fpsyt.2020.00770)
Supplement: Supplementary file 1 [file Table_1.docx]

**Supplementary Table.** Demographic and clinical characteristics of individuals at UHR with and without follow-up

|  | UHR followed up  (N = 24) | UHR without follow-up  (N = 15) | Statistics | |
| --- | --- | --- | --- | --- |
| Sex (Male/Female) ^a^ | 12/12 | 10/5 | χ^2^ = 1.04, df = 1 | p = 0.307 |
| Age (years) ^b, c^ | 20.4 (3.7)  14–28 | 20.9 (4.2)  16–28 | t_37_ = −0.35 | p = 0.728 |
| Education (years) ^b^ | 12.7 (2.6) | 13.0 (2.9) | t_37_ = −0.33 | p = 0.742 |
| Premorbid IQ ^b^ | 105.9 (8.8) | 104.0 (6.6) | t_37_ = 0.70 | p = 0.486 |
| DUPP (days) ^d, e^ | 308.7 (473.8) | 527.6 (696.8) | t_20.7_ = −1.03 | p = 0.314 |
| GAF ^b^ | 47.1 (9.4) | 47.2 (10.8) | t_37_ = −0.04 | p = 0.972 |
| Total SOPS positive subscales ^b^ | 10.9 (3.9) | 9.0 (3.6) | t_37_ = 0.87 | p = 0.391 |
| BACS (z score) ^f^ |  |  |  |  |
| Composite ^b^ | −0.28 (0.91) | −0.50 (0.70) | t_35_ = 0.74 | p = 0.466 |
| Verbal memory ^b^ | −0.33 (1.20) | −1.01 (1.21) | t_35_ = 1.64 | p = 0.110 |
| Working memory ^b^ | −0.33 (1.20) | −0.15 (1.02) | t_35_ = −0.46 | p = 0.647 |
| Motor speed ^b^ | −0.77 (1.56) | −0.80 (1.20) | t_35_ = 0.05 | p = 0.960 |
| Verbal fluency ^d^ | 0.00 (1.48) | −0.31 (0.75) | t_34.9_ = 0.84 | p = 0.407 |
| Attention ^b^ | −0.08 (1.18) | −0.54 (1.06) | t_35_ = 1.16 | p = 0.253 |
| Executive function ^b^ | −0.20 (1.26) | −0.20 (1.13) | t_35_ = 0.005 | p = 0.996 |
| Antipsychotics (mg/day) ^d, g^ | 113.6 (148.7) | 255.1 (429.8) | t_16.1_ = −1.23 | p = 0.236 |
| dMMN amplitude (μV) ^b^ | −1.49 (0.85) | −1.97 (0.71) | t_37_ = 1.83 | p = 0.075 |
| fMMN amplitude (μV) ^b^ | −1.15 (0.57) | −0.97 (0.80) | t_37_ = −0.84 | p = 0.409 |

Legend: All values except for sex are shown as means (standard deviation).

Abbreviations: UHR, ultra-high risk for psychosis; IQ, intelligence quotient; DUPP, duration of untreated prodromal psychosis; GAF, Global Assessment of Functioning; SOPS, Scale of Prodromal Symptoms; BACS, Brief Assessment of Cognition in Schizophrenia; dMMN, duration mismatch negativity; fMMN, frequency mismatch negativity.

^a^ Chi-square test used for statistical comparison.

^b^ Independent t-test used for statistical comparison.

^c^ The range is described in the lower row.

^d^ Welch's t-test used for statistical comparisons.

^e^ Two individuals from the UHR group who were followed up and one from the UHR group without a follow-up have missing DUPP data.

^f^ Two individuals from the UHR group without follow-up have missing BACS data.

^g^ Calculated as chlorpromazine equivalent doses.
